# Indoor Infrared Sensor Layout Optimization for Elderly Monitoring Based on Fused Genetic Gray Wolf Optimization (FGGWO) Algorithm

**DOI:** 10.3390/s24165393

**Published:** 2024-08-21

**Authors:** Shuwang Chen, Yajiang Chen, Meng Feng

**Affiliations:** 1 School of Information Science and Engineering, Hebei University of Science and Technology, Shijiazhuang 050018, China; chenshw@hebust.edu.cn; 2 Department of Acupuncture Hebei Provincial Hospital of Traditional Chinese Medicine, Shijiazhuang 050011, China; mmzz0311@126.com

**Keywords:** elderly monitoring, sensor layout, genetic algorithm, gray wolf optimization algorithm, monitoring system optimization

## Abstract

With the increasing aging of the global population, the efficiency and accuracy of the elderly monitoring system become crucial. In this paper, a sensor layout optimization method, the Fusion Genetic Gray Wolf Optimization (FGGWO) algorithm, is proposed which utilizes the global search capability of Genetic Algorithm (GA) and the local search capability of Gray Wolf Optimization algorithm (GWO) to improve the efficiency and accuracy of the sensor layout in elderly monitoring systems. It does so by optimizing the indoor infrared sensor layout in the elderly monitoring system to improve the efficiency and coverage of the sensor layout in the elderly monitoring system. Test results show that the FGGWO algorithm is superior to the single optimization algorithm in monitoring coverage, accuracy, and system efficiency. In addition, the algorithm is able to effectively avoid the local optimum problem commonly found in traditional methods and to reduce the number of sensors used, while maintaining high monitoring accuracy. The flexibility and adaptability of the algorithm bode well for its potential application in a wide range of intelligent surveillance scenarios. Future research will explore how deep learning techniques can be integrated into the FGGWO algorithm to further enhance the system’s adaptive and real-time response capabilities.

## 1. Introduction

As the global demographic structure changes, the problem of ageing is becoming increasingly serious [1,2]. According to the United Nations, by 2050, the number of people over the age of 65 worldwide will reach 1.5 billion [3]. Due to the frequent occurrence of accidents caused by an unattended home, the home safety of the elderly has gradually become a social problem of global concern [4,5]. An elderly monitoring system is the key to solve this problem [6,7]. As one of the technical means to guarantee the quality of life of the elderly, the effectiveness of the elderly monitoring system is affected by the sensor layout [8]. Sensors are responsible for collecting and detecting key data during elderly monitoring, such as daily life trajectory and health status [9,10]. A reasonable and effective sensor layout plays an important role in significantly improving the overall efficiency and accuracy of the system which can ensure comprehensive monitoring of the daily life trajectory and health status of the elderly [11,12].

However, the sensor layout problem is a multiparameter optimization problem involving factors such as sensor coverage, monitoring continuity, detection efficiency, and cost-effectiveness [13,14]. Current research mainly adopts artificial intelligence and machine learning methods, which are widely used in the optimization of sensor layout to cope with more complex application requirements [15,16]. For example, Song D. and Vitayasak S. et al. proposed genetic algorithms for layout optimization methods [17,18]. Xiu-Wu Y. U. and Kumar S. et al. proposed the use of the wolf pack optimization algorithm for sensor layout optimization [19,20]. Xu Z. and Guo Y. et al. proposed an integrated combinatorial approach based on a reduced-order model and a Gaussian process [21]. Paris R. and Beneddine S. et al. proposed a deep reinforcement learning method [22]. Although these algorithms perform excellently in several application scenarios, they still show limitations with respect to some specific problems. For example, in the study by Loukidis D. et al., it was shown that genetic algorithms, although they have a powerful global search capability, are prone to fall into local optimal solutions, especially in high-dimensional and complex optimization problems [23]. A related study by Wei L. et al. illustrates that wolf pack algorithms are very sensitive to parameter settings during optimization, and an improper parameter selection may lead to a slow convergence or unsatisfactory results [24]. A related study by Zhang R. et al. showed that integrated combinatorial methods for Gaussian processes are highly dependent on model selection and parameter settings, making fine tuning and validation necessary in practical applications [25]. A related study by Vouros G.A. showed that deep reinforcement learning methods rely on a large amount of data and exhibit a significant dependence on computational resources, something which restricts their application in resource-constrained environments, and the process typically lacks transparency and interpretability [26].

In current elderly monitoring systems, installing sensors at key locations in the room, such as near doorways or windows, is often considered an effective option [27,28]. However, it has been shown that sensors in these locations, while capable of capturing human activity, may not be ideal for the comprehensiveness of monitoring [29,30]. In addition, simply increasing the number of sensors to improve monitoring accuracy can pose the same challenges of energy consumption and data processing [31]. Therefore, how to optimize the layout and number of sensors to accurately assess the condition of the elderly while ensuring the quality of the monitoring is an urgent issue [32,33]. In view of this, this study proposes a new algorithm that combines a genetic algorithm and a gray wolf optimization algorithm to optimize sensor layout configurations for elderly monitoring systems using biological evolution and social behavioral mechanisms. The new algorithm combines the powerful global search capability of the genetic algorithm and the efficient local search capability of the gray wolf optimization algorithm, thus effectively avoiding the problem of locally optimal solutions during the iteration process [33,34,35,36]. Through this combination, the sensor locations and numbers can be configured more accurately and efficiently to ensure the comprehensive monitoring of the daily activities of the elderly and achieve a better balance between energy consumption and data processing.

To validate the effectiveness of the algorithm, the experimental design entails the comparison of multiple sensor layout scenarios in a simulated environment and their validation with a real dataset of elderly activities. The experiments evaluate key metrics such as sensor coverage, monitoring accuracy, energy consumption, and data processing efficiency. Through experimental validation, this method demonstrates significant optimization in terms of sensor layout coverage and efficiency, improving the overall performance of the monitoring system. Meanwhile, potential challenges and limitations are also discussed in this study, with a view to providing references and improvement directions for future research. This study is not only theoretically significant, but it also has a wide range of prospects for practical applications, especially in smart homes and medical monitoring systems. Through experimental validation, this method exhibits significant optimization success in terms of coverage and efficiency of sensor layout, enhancing the overall performance of the monitoring system.

## 2. FGGWO Algorithm for Indoor Infrared Sensor Layout

This paper tries to find the most suitable sensor layout for detecting the activities of elderly people in indoor places in order to accurately predict their condition. The proposed method is applicable to general indoor places.

We assume that the indoor environment is divided into *n* work zones based on its function and physical layout, where each work zone has sensors for monitoring the behavior of the elderly to ensure that the monitoring system can fully cover and effectively respond to the activity needs of the elderly. Therefore, the activity frequency and activity status of the elderly within the work zones are key considerations in the layout design [37]. Based on this, the indoor infrared sensor layout of the elderly monitoring system is designed to accurately and effectively monitor the condition of the elderly. In order to achieve the above monitoring objectives, the sensor layout of the activity area needs to be determined. By accurately determining the layout of the elderly’s main activity paths and commonly used areas, it is possible to use the minimum number of sensors to accurately reflect the intensity of the elderly’s activities and behavioral anomalies. In addition, in order to avoid interference from environmental factors such as light variations and indoor heat sources, each work area needs to take into account the influence of these external factors. This requires a comprehensive design approach that takes into account both the activity characteristics of the elderly and the environmental factors, thus ultimately minimizing monitoring blind spots and improving the comprehensiveness of the monitoring.

### 2.1. FGGWO Algorithm

**Figure 1:**
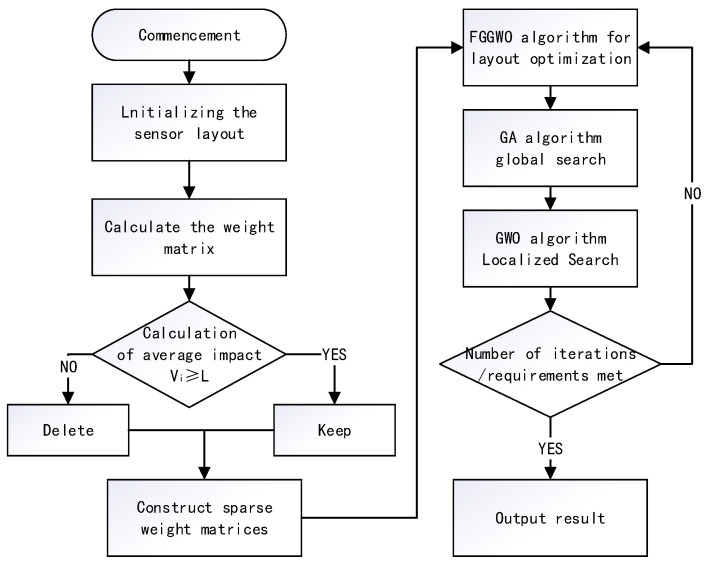
Optimization flowchart for the FGGWO algorithm to determine the sensor layout in the detection area.

For an indoor environment using *z* sensors, the optimization flowchart of the FGGWO algorithm to determine the sensor layout in the detection area is shown in Figure 1.

Firstly, *h* sensors were evenly placed in the work area, the activity intensity of the elderly within the monitoring range of each sensor was measured, and an initial weight matrix was created based on these data. Next, the weight matrix was thinned to reduce the computational complexity. Finally, the best sensor locations were filtered by the FGGWO algorithm to ensure the optimization of the sensor layout.

#### 2.1.1. Weighting Matrix

The indoor environment was divided into work zones and each work zone was divided into a grid-like shape. Each workspace was divided by introducing a vector *P* in order to detect and record the activity data of the elderly. The vector *P* is described in Equation (1).

(1) $$P_{i}^{t}=\left[P_{1}^{t}\begin{array}{ccc} P_{2}^{t} & \ldots & P_{h}^{t} \end{array}\right]$$

where *P^t^* denotes the *h* sensors in the *t*th work zones. In this setup, each sensor can cover multiple grid cells.

The range of the elderly and the intensity of their activities affect the sensors differently and, depending on the relative distance between the elderly and the sensors, the lightness of the signals received by the sensors is different. In order to quantify the degree of influence of the elderly’s activity on the sensor, we introduced an attenuation coefficient α and defined the weight $W_{\mathrm{ij}}$ to reflect the relationship between the influence of the elderly’s activity on the sensor within the detection range of the *j*th sensor. The weights were calculated as in Equation (2).

(2) $$W_{ij}=\frac{e^{-\alpha d_{ij}}}{\sum_{k=1}^{h}e^{-\alpha d_{ik}}}$$

where $e^{-\alpha d_{ij}}$ denotes an exponential attenuation term that describes the signal attenuation with increasing distance $d_{ij}$, $d_{ij}$ is the distance from the *i*th sensor to the target elder, α denotes an attenuation coefficient that controls the rate at which the distance affects the attenuation, h denotes the total number of sensors, and $\sum_{k=1}^{h}e^{-\alpha d_{ik}}$ denotes a normalization factor, which is the sum of the attenuations of the *i*th sensor relative to all the targets and makes sure that the sum of the weights is 1. The weights $W_{\mathrm{ij}}$ provide a way to measure the influence of the elderly on the sensors.

In addition, we considered the total number of activity frequencies $l_{c}$ of the elderly in the indoor environment and calculated the weight of the impact of each activity on each sensor in the workspace to form the weight matrix W, where each row of the weight matrix W corresponds to the influence weight of an activity on all sensors and each column reflects the combined influence of a particular sensor on all activities. Each element $W_{\mathrm{ij}}$ represents the influence weight of the *i*th sensor on the *j*th activity. This weight calculation takes into account the total number of activity frequencies for older adults and extends it in the time dimension.

Through the matrix representation, we were able to accurately quantify the contribution of the sensors to the detection of elderly activities, thus providing a quantitative basis for optimizing the sensor layout. In addition, considering the dynamic changes and potential interference in the environment, the model can be adjusted to adapt to different indoor environmental conditions by adjusting the attenuation coefficient α, which enhances the practicality of the model.

#### 2.1.2. Sparse Weight Matrices

For indoor sensor layout design, since some sensors in the workspace are less sensitive to monitoring activities, we introduced the sparse matrix $W^{*}$, which is designed to optimize the performance of the monitoring system by filtering out those sensors that exhibit lower sensitivity when monitoring the activities of the elderly. This strategy not only reduces the computational burden, but also improves the overall response speed and accuracy of the system. Specifically, the sparse matrix $W^{*}$ is constructed by evaluating the contribution of each sensor to the monitoring activity and by removing those sensors whose contribution is below a preset threshold. To this end, we first defined an evaluation metric that integrated the distance between the elderly and the sensors as well as the attenuation effect α of the distance to measure the sensitivity of the sensors to monitor the activities of the elderly.

Assuming that the changes in sensor values caused by the activities of the elderly are recorded in *s* test cases, the average impact value $V_{i}$ of the *i*th sensor can be calculated by using the following Equation (3).

(3) $$V_{i}=\frac{1}{S}\sum_{k=1}^{s}\frac{\left|\Delta P_{i,k}\right|}{d_{i,k}}$$

where $\Delta P_{i,k}$ denotes the change in value of the *i*th sensor in the *k*th test case due to the activity of the elderly, $d_{i,k}$ denotes the distance between the *i*th sensor and the location of the elderly activity, and α is the attenuation coefficient, which is used to adjust the effect of distance on the sensitivity of the sensor response. By adjusting the effect of distance, we ensure that the response sensitivity of the sensors decreases appropriately with the increase in distance, so that the actual interaction effect between the elderly and the sensors can be reflected more accurately.

Initially, we collected data from sensors deployed throughout the monitored space, not just within a single workspace. This was done to ensure that the data we collected would provide a comprehensive picture of the space usage as well as the necessary basis for constructing the weighting and sparsity matrices. In practice, where direct data from a dense array of sensors were not be available, we used simulated or generic motion data to estimate these effects, an approach that ensures that the algorithm works even when the initial data are incomplete and that the algorithm becomes progressively more effective as the data accumulate and the system is tuned.

Based on the evaluation metrics, a sparse matrix, $W^{*}$, was further constructed. Each element $W_{ij}^{*}$ of this matrix reflects the sensitivity of the *j*th sensor to monitor the *i*th activity, calculated as in Equation (4).

(4) $$W_{ij}^{*}=\frac{1}{d_{ij}^{\alpha }}$$

where $d_{ij}$ is the distance between the location of the *i*th event and the *j*th sensor.

Next, a threshold L was set to determine which sensor impact weights could be ignored as insignificant. If the average influence value $V_{i}$ of a sensor was less than L, the corresponding weight in the sparse matrix $W^{*}$ would be 0 or removed from the matrix; if $V_{i}$ was greater than L, the corresponding weight in the sparse matrix would be retained, as shown in Equation (5).

(5) $$W^{*}=\{\begin{array}{ll} W_{\mathrm{ij}}^{*} & \mathrm{if},W_{\mathrm{ij}}^{*}\geq L\\ 0 & \mathrm{if},W_{\mathrm{ij}}^{*}\leq L \end{array}$$

The threshold L plays a key role in the design of indoor sensor layouts. Its selection is based on a preformed sensitivity analysis to ensure that its impact on the simulation results can be accurately assessed, and the setting of L directly affects the computational complexity and monitoring accuracy of the system. Therefore, setting a larger value of L at the beginning of the optimization procedure helps quickly simplify the sensor network and reduce the number of sensors initially considered. With repeated iterations of the optimization process, the threshold value was gradually lowered to refine the selection of sensors and enhance the system’s ability to monitor critical areas until the desired monitoring results were achieved. In this study, the initial threshold value was set to L=1 and was gradually increased or decreased according to the needs of the simulation process until a suitable value was found to ensure the stability and accuracy of the results. Our results are based on the comprehensive evaluation of this series of L values which demonstrates the specific impact of parameter variations on monitoring effectiveness.

With this approach, the weight elements retained in the resulting sparse weight matrix $W^{*}$ all represent sensors that have a high impact on the system. This means that the non-zero weight values in the matrix point to those sensor locations that are most sensitive to the monitoring of the elderly’s activities, thus ensuring that the highest monitoring efficiency is achieved using the minimum number required of sensors. This sparse weight matrix $W^{*}$ was used as input data for the Fusion Genetic Algorithm and Gray Wolf Optimization (FGGWO) algorithm to select the optimal sensor layout.

#### 2.1.3. FGGWO Combination Algorithm

In order to select the optimal sensor layout, i.e., to achieve the minimum number of sensors required as well as maximize the monitoring efficiency, the FGGWO algorithm, a combination of Genetic Algorithm (GA) and Gray Wolf Optimization (GWO) algorithm, was used in this study. This combined algorithm takes advantage of the characteristics of GA’s global search ability in space and GWO’s fast local search ability. Combining the advantages of both, the FGGWO algorithm establishes an efficient optimization framework that can effectively balance the relationship between exploration and exploitation, improve the efficiency and accuracy of finding the optimal solution, and can more objectively reflect the optimal configuration of the sensor layout in a complex environment.

##### Genetic Algorithm Part

The Genetic Algorithm (GA) is a search algorithm that mimics the principles of natural selection and genetics in biological evolution. It solves optimization problems through the concepts of population, heredity, and selection [38]. In indoor sensor layouts, each layout scheme can be considered as an ‘individual’, and each individual consists of a specific set of sensor positions (i.e., a chromosome). The main steps of the GA include selection (picking the best individual), crossover (mixing the features of two individuals to generate a new one), and mutation (randomly changing some features of an individual to explore a new solution space). The genetic algorithm evaluates the fitness (i.e., coverage and monitoring efficiency) of each layout and generates a better layout solution through genetic operations. These operations ensure the diversity of the population and the comprehensiveness of the solutions.

##### Gray Wolf Optimization Algorithm Section

The Gray Wolf Optimization Algorithm (GWO) simulates the social hierarchy and hunting strategy of a wolf pack to find a globally optimal solution [39]. In the algorithm, the leader of the pack (alpha, beta, and delta) is used to guide the rest of the pack (omega) to update its position so as to be close to the prey, the optimal solution of the problem. This algorithm excels in local search and fast convergence and is suitable for refining the sensor layout that has been initially determined by the genetic algorithm.

##### FGGWO Combined Algorithm

In order to make full use of the respective advantages of the Genetic Algorithm (GA) and the Gray Wolf Optimization (GWO) algorithm, the FGGWO algorithm firstly performs a global search through the genetic algorithm to determine an optimal sensor layout. Subsequently, the gray wolf optimization algorithm performs a local search and an optimization based on this layout to improve the accuracy and efficiency of the layout. The objective function of the FGGWO algorithm is defined as shown in Equation (6).

(6) $$F\left(x\right)=\alpha \cdot Coverage\left(x\right)+\beta \cdot Efficiency\left(x\right)+\gamma \cdot S\mathrm{ensitivity}\left(x\right)$$

where x denotes a particular sensor layout scheme, $Coverage\left(x\right)$ denotes the coverage of that layout scheme, $Efficiency\left(x\right)$ denotes the monitoring efficiency, $S\mathrm{ensitivity}\left(x\right)$ denotes the sensitivity of the sensor, and α, β, and γ are weighting coefficients used to adjust the relative importance of different performance indicators in the objective function, with the specific values needing adjustment according to different application scenarios and focuses. For example, in scenarios requiring high coverage, a larger α value can be chosen, while in applications emphasizing efficiency, the weight of β can be increased. In cases where sensitivity is more critical, the weight of γ can be increased. The specific selection can be adjusted according to the experimental data to achieve the best monitoring effect.

This objective function needs to consider not only the actual impact of these performance metrics, but also the trade-offs between them. In order to find the optimal solution to the objective function, the key mathematical conditions of the optimization process must be more specifically defined and highlighted, as in Equations (7) and (8). This allows the algorithm to follow the right direction during the optimization process, balancing multiple metrics, so that the objective function gradually converges to the optimal solution.

(7) $$\frac{\partial F\left(x\right)}{\partial x_{i}}=0,\;\;\;for\;all\;i$$

(8) $$L\left(x,\lambda \right)=F\left(x\right)+\lambda \cdot \left(g\left(x\right)-c\right)$$

The FGGWO algorithm is divided into two main steps: initialization and iterative computation. Firstly, it is necessary to determine the initialization phase including setting the algorithm parameters such as population size, crossover rate, mutation rate, and generating the initial population. Subsequently, the algorithm enters the iterative computation phase, where each iteration includes GA selection, crossover, and mutation operations, and GWO position update. Through the iterative optimization process, the FGGWO algorithm gradually improves the sensor layout until a predefined threshold or maximum number of iterations is reached, at which point the algorithm stops.

The GA operation updates the population through Equation (9) [40].

(9) $$x^{\prime }=crossover\left(mutate\left(select\left(Pop\right),p_{m}\right),p_{c}\right)$$

where select, crossover, and mutate represent the selection, crossover, and mutation operations and $p_{m}$ and $p_{c}$ are mutation and crossover probabilities, respectively.

GWO, then, adjusts the position of the other individuals according to the position of the optimal individual in the current population, updating the formula in Equation (10) [41].

(10) $$X_{new}=X_{alpha}-A\cdot D+C\cdot \left(X_{beta}-X_{gamma}\right)$$

where $X_{alpha}$, $X_{beta}$, and $X_{gamma}$ are the positions of the lead wolf, A and C are coefficients controlling the search intensity and randomness, and D is a vector of distances between the wolf and the target [42].

Through the iterative optimization process described above, iterations are performed until the termination condition is satisfied, i.e., when the change in the objective function between two consecutive generations, $\Delta F=\left|F\left(x_{new}\right)-F\left(x_{old}\right)\right|$, is less than a preset threshold, L, or until the maximum number of iterations, $N_{\max}$, is reached.

##### Processing of Weight Matrices

Next, a combination of the weight matrix W and the sparse matrix $W^{*}$ was used to process the weights of each sensor’s impact on the monitored area in the sensor layout. This was to obtain a cost-effective and efficient sensor layout scheme that can accurately reflect the measurement needs of each region and the effectiveness of the sensors.

Each element $W_{ij}$ of the weight matrix W represents the degree of influence of the *ith* sensor on the *jth* region, which is usually based on the distance of the sensor from the region as well as on the performance parameters of the sensor. As not all sensors are equally important to each region, this process was optimized by creating a sparse matrix $W^{*}$ containing only the significant weights, i.e., those whose influence is significantly above a certain threshold. Such a process reduces computational complexity and allows the algorithm to focus its resources on optimizing those sensor configurations that have the greatest impact on the monitoring results.

The key to using the FGGWO algorithm is to set the correct weighting coefficients α, β, and γ and control parameters (e.g., coefficients A and C in the gray wolf algorithm and the crossover rate $p_{c}$ and the mutation rate $p_{m}$ in the genetic algorithm). Excessively small weighting coefficients can lead to inaccurate priority judgements, resulting in certain key performance indicators being ignored, while excessively large control parameters can lead to excessive resource consumption, in turn leading to waste. Therefore, the merit of the algorithm effect is evaluated using the following performance evaluation equation, Formula (11).

(11) $$Performance\left(x\right)=\frac{1}{N}\sum_{i=1}^{N}\left(\lambda_{1}\cdot C\left(i,x\right)+\lambda_{2}\cdot S\left(i,k\right)+\lambda_{3}\cdot E\left(i,k\right)\right)$$

where N denotes the total number of monitored areas, each of which may be covered by one or more sensors, x denotes a particular sensor layout scheme, $C\left(i,x\right)$ denotes the coverage of the sensor in the *i*th area, $E\left(i,x\right)$ denotes the efficiency of the sensor in the *i*th area, $S\left(i,x\right)$ denotes the sensitivity of the sensor in the *i*th area, and $\lambda_{1}$, $\lambda_{2}$, $\lambda_{3}$ are weighting coefficients representing the relative importance of coverage, sensitivity, and efficiency, respectively, in the performance evaluation. The selected parameters are adjusted according to the actual situation when the objectives and requirements are different at different stages.

The algorithm is initiated after setting the threshold L=1 to eliminate the sensors whose efficiency or coverage is lower than this threshold. If the weight matrix W has *N* rows, then *N* sensors are finally selected. The final retained sensors are the sum of the sensors with the largest weights in each row, and the number and location of sensors obtained at this point is also the recommended configuration. Then, the performance evaluation value of the recommended configuration is calculated and, if the evaluation value is not 0, the threshold continues to be fine-tuned until the performance requirement is met. Using the minimum number of active sensors will help reduce the computation as well as the workload, and the accuracy of the final control system will not be significantly reduced compared to the initial configuration.

This evaluation methodology provides a comprehensive measure of the performance of sensor layout solutions in real-world monitoring applications and is designed to support the optimization of solutions to improve their feasibility, cost-effectiveness, and efficiency. This evaluation criterion helps motivate the FGGWO algorithm to find the most suitable sensor layout solution in various monitoring scenarios.

## 3. Experimental and Discussion

### 3.1. Experimental Conditions

Of the many different room configurations tested, this paper provides representative room layout configurations and zone arrangements. Our study takes into account the correlation among the activity patterns of the elderly, the layout of the room furniture environment, and the sensor layout, and rationalizes the sensor layout by analyzing the design characteristics of the room so as to maximize the coverage efficiency in the existing environment. This process is not a simple assumption but, rather, it is based on a detailed examination of room-specific characteristics and layout optimization. The detection surfaces were selected to cover all key activity areas (e.g., living room, kitchen, bedroom, and bathroom, etc.), and the space occupied by the sensors needed to be minimized, with all the sensors mounted at a height of 1.2 m above the floor and the mounting angle adjusted to maximize the coverage of the key activity areas, while avoiding the creation of blind spots and achieving the effect of not being conspicuous and not hindering daily activities. The room details are shown in Table 1 below.

**Figure 2:**
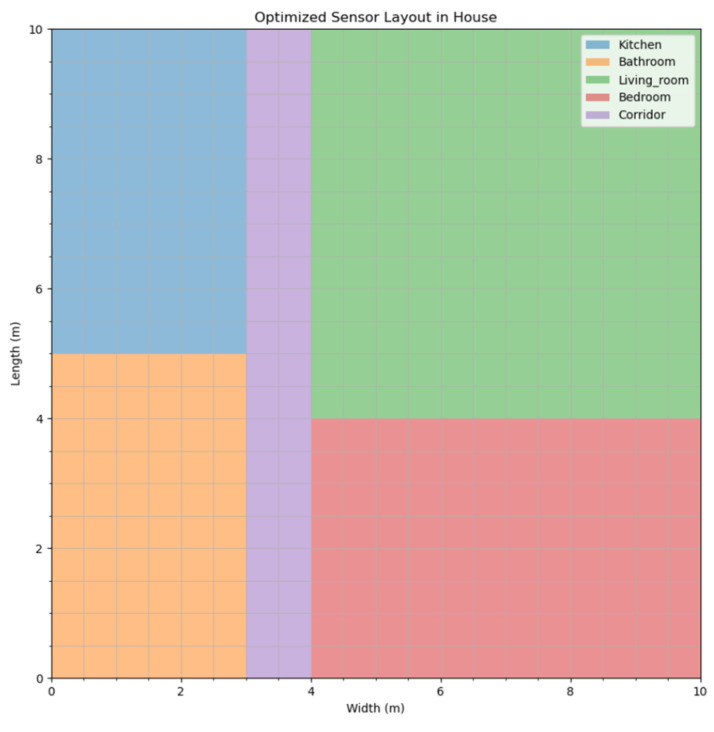
Room area division.

Data on the living habits of multiple groups of elderly people, five in total, were selected for the experiment, with each group focusing on a different life trajectory, thus ensuring that the test could cover a wide range of activity patterns and possible scenarios. A series of daily activities such as waking up, eating, resting, and performing indoor exercises were performed in the same room environment for each group to simulate the dynamics of a real home environment. The next layout experiment was conducted in this combined scenario with the aim of optimizing the sensor layout to maximize monitoring effectiveness and efficiency. The scenario consisted of multiple work areas such as living room, kitchen, bedroom, and bathroom to ensure that every important living space was taken into account. As shown in Figure 2, these work areas were divided to match different types of activities and the specific needs of the elderly.

### 3.2. Data Acquisition

In carrying out the collection and processing of the data on the activities of the elderly, five groups of elderly people were used as study subjects, each with different habits and activity patterns to ensure that the study covered a wide range of scenarios. Data collection was mainly conducted through infrared sensors installed at key locations in the room and the characteristics of the data collected are shown in Table 2. Infrared sensors are capable of detecting infrared radiation emitted by the human body, thus monitoring the movements and health status of the elderly.

### 3.3. Infrared Sensor Layout

In this study, we chose a Passive Infrared (PIR) sensor to monitor the daily activities of elderly people. The PIR sensor senses the activity status of an elderly person by detecting infrared radiation emitted by the human body, as well as human movement. The main advantages of this sensor include a high cost-effectiveness, a low power consumption, and the protection of user privacy by not capturing specific images, making it ideal for long-term monitoring of elderly activities. When considering other monitoring technologies such as Wi-Fi radar and Doppler radar, we found that the monitoring accuracy of the Wi-Fi radar may be interfered with by the surrounding environment and that such a radar is particularly ineffective in environments with multiple devices interfering with it, while the Doppler radar is unsuitable for older adult monitoring due to its higher cost and potential privacy invasion issues. Therefore, after synthesizing the cost, privacy protection, and monitoring needs, we finally chose the PIR sensor as the monitoring device in this study.

**Figure 3:**
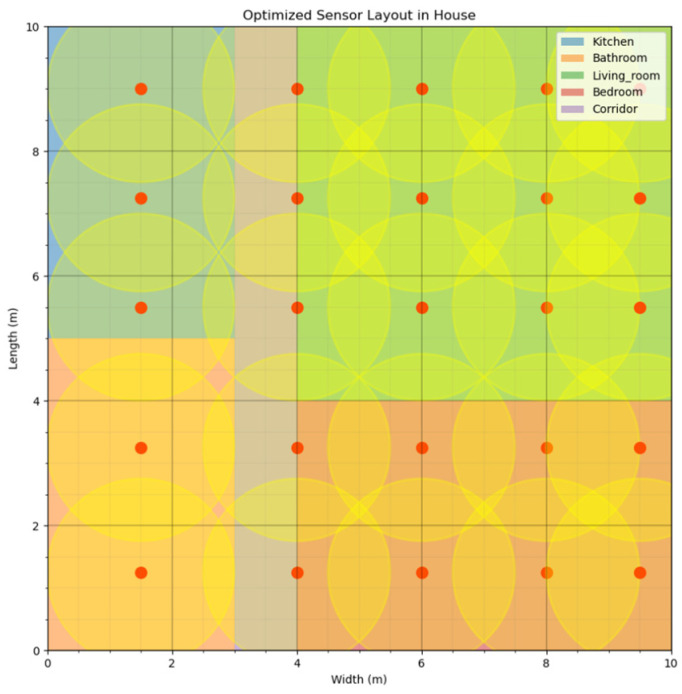
Schematic diagram of the position of the initial sensor placement.

The initial deployment of PIR sensors was not intended to determine the final or optimal layout for monitoring the daily activities of older adults. As shown in Figure 3, initially, a large number of sensors was evenly distributed throughout the room to comprehensively collect environmental data. These data were then used as input for the FGGWO algorithm, which optimizes the sensor layout based on actual activity patterns and environmental feedback. This approach helps the algorithm adjust and refine the layout based on actual usage.

**Figure 4:**
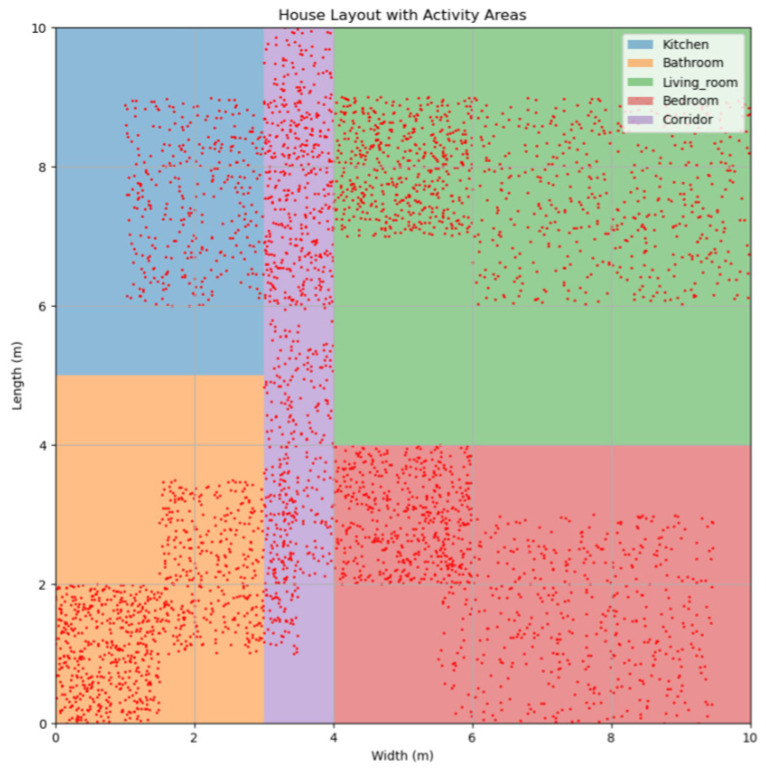
Schematic diagram of the elderly activity areas in the room.

Firstly, by collecting data on the daily activities of the elderly, the frequency of activities in each area of the room was assessed to determine the weights of these areas. As shown in Figure 4, the areas experiencing frequent elderly activity are displayed.

Next, a weighting matrix was calculated based on the collected activity data and a predetermined attenuation factor. This matrix reflects the relative importance of each area with respect to the monitoring system, eliminating areas that have a lesser impact on the sensors. The results are shown in Table 3. This step helps reduce the number of redundant sensors by determining the size of the sparse matrix. The size of the sparse matrix reflects the actual monitoring efficiency and cost-effectiveness of the optimized sensor network. This approach helps overcome a redundant configuration of sensors while ensuring the accuracy and efficiency of the monitoring system.

Through the above steps, we were able to accurately assess the importance of each area with respect to the monitoring system so as to optimize the sensor layout, reduce redundant sensors, and ensure the efficiency and accuracy of the system.

The obtained sparse matrix was used as the input data for the FGGWO algorithm, which first recalculates the individuals of the population based on the weights, generates the individuals of the initial population, and proposes a new preliminary sensor layout scheme. Subsequently, local optimization was performed using the Gray Wolf Optimization (GWO) algorithm to fine-tune the sensor locations based on the fitness scores of the individuals in the population in order to improve the accuracy and coverage of the sensor layout. In this process, the algorithm determines the optimal sensor layout scheme through a series of iterations until the set convergence conditions or the maximum number of iterations is reached, a process which effectively improves the efficiency of the sensors and the accuracy of the data.

### 3.4. Analysis of Results

**Figure 5:**
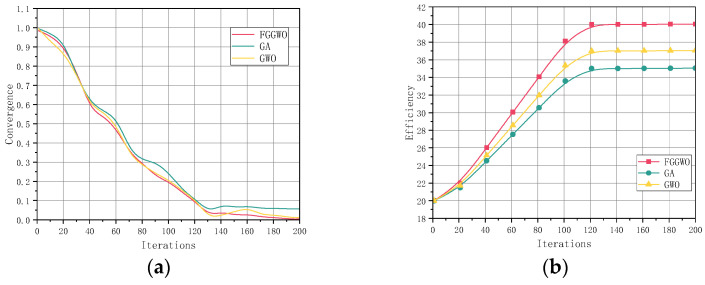
(**a**) Comparison of convergence speed of three algorithms; (**b**) comparison of efficiency of three algorithms.

Convergence and efficiency were the main metrics used to evaluate the performance of the algorithms. Each algorithm underwent 200 iterations to calculate the convergence as well as the efficiency. From the results in Figure 5, we can see that the FGGWO algorithm demonstrated a faster convergence and a higher running efficiency. The convergence of the FGGWO algorithm decreased rapidly and eventually approached 0, indicating that it was more effective in finding the optimal solution, while its running efficiency gradually improved to close to 40. In contrast, the GA and GWO algorithms converged more slowly, with the convergence eventually remaining at a high level, and the operational efficiency stabilized at around 30 and 35, respectively. The values of convergence and efficiency indicate that the FGGWO algorithm was able to approach the optimal solution more rapidly, while being more efficient in terms of resource utilization and running time.

**Figure 6:**
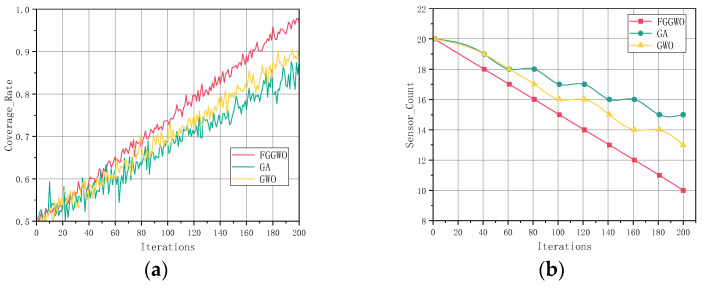
(**a**) Comparison of coverage of three algorithms; (**b**) comparison of number of sensors of three algorithms.

Coverage is a key measure of the effectiveness and comprehensiveness of the sensor layout. The optimal sensor layout coverage of the three algorithms is shown in Figure 6. As it can be seen from Figure 6, the FGGWO algorithm layout attained the highest coverage with the same number of sensors. In addition, the results show that the FGGWO algorithm significantly reduced the number of sensors during the iterative process, and the final number of sensors stabilized at around 10, while the coverage rate rapidly increased to close to 100%, demonstrating high efficiency. In contrast, the GA and GWO algorithms reduced the number of sensors and improved the coverage more slowly, and eventually stabilized at about 15 and 13 sensors, respectively, with coverage reaching about 85% and 90%, respectively. In Figure 6b, showing the comparison of the number of sensors of three algorithms, the reduction of sensors is not uniformly linear but phased. The number remains stable at a particular iteration stage and, then, it rapidly decreases to a new stable value at the next iteration point until the coverage area requirement is satisfied. This suggests that the FGGWO algorithm is able to provide a wider monitoring coverage under the same resource conditions, thus ensuring the efficacy and reliability of the monitoring system.

By comparing the comprehensive performance of the different algorithms, we can see where the FGGWO algorithm excelled. The FGGWO algorithm not only performed well in sensor coverage, but it also showed significant advantages in algorithm convergence and operation efficiency, details of which are shown in Table 4. This makes the FGGWO algorithm a more desirable solution. These results highlight the potential of the FGGWO algorithm for application in sensor layout optimization based on the experimental results obtained.

In comparing the experimental results from the three algorithms, FGGWO, GA, and GWO, we analyzed the results in detail with respect to five aspects: the number of sensors, coverage, convergence, efficiency, and running time. The results show that the FGGWO algorithm significantly reduces the number of sensors during the iterative process and eventually stabilizes at around 10, while the coverage rate rapidly increases to close to 100%, demonstrating high efficiency. In contrast, the GA and GWO algorithms reduce the number of sensors and improve the coverage more slowly, eventually stabilizing at around 15 and 13, respectively, with a coverage of around 85% and 90%, respectively. In addition, the FGGWO algorithm converges quickly, the convergence decreases rapidly to nearly 0, the operation efficiency gradually increases to nearly 40, and the shorter operation time exhibits higher efficiency. In summary, the FGGWO algorithm demonstrates excellent performance in sensor layout optimization, including higher efficiency, coverage, fast convergence, shorter running time, and lower energy consumption, suggesting that it has a high application potential and practical value in sensor layout optimization.

### 3.5. Comprehensive Comparative Analysis

In order to comprehensively evaluate the performance and applicability of the FGGWO algorithm in elderly monitoring systems, this section compares it with other major algorithms in the field. Through this comparison, we can see more clearly the advantages of the FGGWO algorithm in specific application scenarios.

In elderly surveillance systems, the optimization of sensor layout is crucial for improving system performance and monitoring effectiveness. The IALSCC algorithm and the metaheuristic algorithms, which are widely used in current research, have limitations with respect to targeting the specific needs of elderly monitoring systems despite their excellent performance in several other application areas. The IALSCC algorithm, as pointed out in the literature [43], is mainly applicable to the health monitoring of large structures, with the optimization objective focusing on the stability and durability of the engineered structure, while the algorithm is insufficiently adapted to the complexity and nonlinear characteristics of the indoor behavior of the elderly. The metaheuristic algorithms discussed in the literature [44], although they can effectively improve the coverage and connectivity in the layout optimization of wireless sensor networks, usually lack the focus on the accurate monitoring of specific behaviors of the elderly, especially with respect to applications in variable indoor environments.

Compared with these methods, the proposed FGGWO algorithm is designed for the indoor environment monitored by elderly monitoring systems, which combine the global search advantage of the genetic algorithm and the local search accuracy of the gray wolf optimization algorithm, effectively overcoming the shortcomings of the above algorithms. In the experiments presented in this paper, the FGGWO algorithm showed its significant advantages with regard to coverage, convergence speed, and optimization of the number of sensors. Compared with the traditional genetic algorithm and the gray wolf optimization algorithm, the FGGWO algorithm not only provides higher coverage, but also achieves higher efficiency and accuracy in sensor configuration, clearly demonstrated in Figure 5 and Figure 6.

Compared with other algorithms, the FGGWO algorithm has the advantage of being specialized and adaptable in its design and of overcoming the limitations of traditional methods. In this paper, we not only studied the daily behavioral patterns of the elderly, but also integrated these behavioral characteristics into the design of the algorithm to ensure higher monitoring accuracy and system performance in practical applications.

## 4. Conclusions

In this paper, we propose the study of the optimization of sensor layouts in elderly monitoring systems by adopting the sensor layout optimization algorithm, the FGGWO algorithm, which is a combination of the genetic algorithm and the gray wolf optimization algorithm. The results demonstrate that the FGGWO algorithm can significantly improve the coverage and efficiency of the monitoring system and reduce the number of required sensors when optimizing sensor layouts compared to the more traditional methods while maintaining high-precision activity monitoring, showing the potential and benefits of optimization algorithms in dealing with complex system configuration problems. Compared with the IALSCC algorithm and the metaheuristic-based wireless sensor network layout optimization technique described in the literature, the FGGWO algorithm demonstrates its unique advantages in specific application scenarios. Therefore, the innovation of the FGGWO algorithm is that it is specifically optimized for elderly monitoring systems, something which makes it not only technically adapted to specific application scenarios, but also results in higher cost-effectiveness and wide applicability in practice. These features make the FGGWO algorithm a cost-effective solution in the field of smart home and elderly health monitoring, with the potential to be extended to other types of monitoring systems. Future research could extend the algorithm for application in other types of monitoring systems and evaluate the integration of deep learning techniques to improve the system’s adaptive and real-time responsiveness. Through these studies, we expect to provide a safer and more automated home monitoring environment for the elderly.

## Figures and Tables

**Table 1:** Room-specific details.

Area	Dimensions	Layout	Usage
Kitchen	3 m × 5 m	Includes kitchen island, hob, fridge, cupboards	Cooking, dining
Bathroom	3 m × 5 m	With bathtub, shower cubicle, washbasin, toilet	Bathing, daily hygiene
Living room	6 m × 6 m	With sofa, coffee table, TV cabinet, bookshelf	Daily activities, resting
Bedroom	6 m × 4 m	With double bed, wardrobe, desk, dressing	Resting, private activities
Hallway	1 m × 10 m	None	Walking access

**Table 2:** Table of data collection characteristics (percentage of time in each area).

Grouping	Living Room	Bedroom	Bathroom	Kitchen
Elderly group 1	44	37	7	12
Elderly group 2	39	42	8	11
Elderly group 3	46	41	5	8
Elderly group 4	44	34	10	12
Elderly group 5	39	44	6	11

**Table 3:** Table describing the weighting of the various regions.

Area Index	Position (x, y)	Size (Width × Length)	Weight
1	(0, 0)	2 × 1.5	High
2	(1, 1.5)	2.5 × 2	Medium
3	(0, 5.5)	3 × 4	High
4	(2, 3)	8 × 1	Low
5	(2, 4)	2 × 2	Medium
6	(6, 1)	3 × 3	Medium
7	(6, 6)	3 × 4	High
8	(7, 4)	2 × 2	Low

**Table 4:** Comparison table of integrated performance.

Arithmetic	Sensors	Coverage	Convergence	Efficiency	Runtime
FGGWO	10	0.97255	0.01615	40.00360	5999.87786
GA	15	0.85975	0.06142	35.01885	5499.49618
GWO	13	0.90104	0.03965	37.01108	5699.83758

## Data Availability

Data underlying the results presented in this paper are not publicly available at this time but may be obtained from the authors upon reasonable request.

## References

[1] U.N.ESCAP (2022). Asia-Pacific Report on Population Ageing 2022: Trends, Policies and Good Practices Regarding Older Persons and Population Ageing. https://repository.unescap.org/handle/20.500.12870/4963.

[2] Bloom D.E., Luca D.L. (2016). The global demography of aging: Facts, explanations, future. Handbook of the Economics of Population Aging.

[3] Padeiro M., Santana P., Grant M. (2023). Global aging and health determinants in a changing world. Aging.

[4] Kivimäki T., Stolt M., Charalambous A., Suhonen R. (2020). Safety of older people at home: An integrative literature review. Int. J. Older People Nurs..

[5] Cantone A.A., Esposito M., Perillo F.P., Romano M., Sebillo M., Vitiello G. (2023). Enhancing Elderly Health Monitoring: Achieving Autonomous and Secure Living through the Integration of Artificial Intelligence, Autonomous Robots, and Sensors. Electronics.

[6] Majumder S., Aghayi E., Noferesti M., Memarzadeh-Tehran H., Mondal T., Pang Z., Deen M.J. (2017). Smart Homes for Elderly Healthcare—Recent Advances and Research Challenges. Sensors.

[7] Diraco G., Leone A., Siciliano P. (2017). A Radar-Based Smart Sensor for Unobtrusive Elderly Monitoring in Ambient Assisted Living Applications. Biosensors.

[8] Kekade S., Hseieh C.H., Islam M.M., Atique S., Khalfan A.M., Li Y.C., Abdul S.S. (2018). The usefulness and actual use of wearable devices among the elderly population. Comput. Meth. Programs Biomed..

[9] Ahmed S., Irfan S., Kiran N., Masood N., Anjum N., Ramzan N. (2023). Remote Health Monitoring Systems for Elderly People: A Survey. Sensors.

[10] Uddin M.Z., Khaksar W., Torresen J. (2018). Ambient sensors for elderly care and independent living: A survey. Sensors.

[11] Wang Y., Chen Y., Yao Y., Ou J. (2023). Advancements in Optimal Sensor Placement for Enhanced Structural Health Monitoring: Current Insights and Future Prospects. Buildings.

[12] Hassani S., Dackermann U. (2023). A systematic review of advanced sensor technologies for non-destructive testing and structural health monitoring. Sensors.

[13] Hassani S., Dackermann U. (2023). A Systematic Review of Optimization Algorithms for Structural Health Monitoring and Optimal Sensor Placement. Sensors.

[14] Bidar O., Anderson S.R., Qin N. (2024). Sensor placement for data assimilation of turbulence models using eigenspace perturbations. Phys. Fluids.

[15] Seaman K., Ludlow K., Wabe N., Dodds L., Siette J., Nguyen A., Jorgensen M., Lord S.R., Close J.C., O’Toole L. (2022). The use of predictive fall models for older adults receiving aged care, using routinely collected electronic health record data: A systematic review. BMC Geriatr..

[16] Anitha G., Priya S.B. (2022). Vision Based Real Time Monitoring System for Elderly Fall Event Detection Using Deep Learning. Comput. Syst. Sci. Eng..

[17] Vitayasak S., Pongcharoen P., Hicks C. (2017). A tool for solving stochastic dynamic facility layout problems with stochastic demand using either a Genetic Algorithm or modified Backtracking Search Algorithm. Int. J. Prod. Econ..

[18] Song D., Yan J., Zeng H., Deng X., Yang J., Qu X., Rizk-Allah R.M., Snášel V., Joo Y.H. (2023). Topological Optimization of an Offshore-Wind-Farm Power Collection System Based on a Hybrid Optimization Methodology. J. Mar. Sci. Eng..

[19] Wu Y., Hao Y., Yong L., Rong X. (2020). A clustering routing algorithm based on wolf pack algorithm for heterogeneous wireless sensor networks. Comput. Netw..

[20] Kumar S., Kumar H., Kumar G., Singh S.P., Bijalwan A., Diwakar M. (2024). A methodical exploration of imaging modalities from dataset to detection through machine learning paradigms in prominent lung disease diagnosis: A review. BMC Med. Imag..

[21] Xu Z., Guo Y., Saleh J.H. (2022). Multi-objective optimization for sensor placement: An integrated combinatorial approach with reduced order model and Gaussian process. Measurement.

[22] Paris R., Beneddine S., Dandois J. (2021). Robust flow control and optimal sensor placement using deep reinforcement learning. J. Fluid Mech..

[23] Aivaliotis-Apostolopoulos P., Loukidis D. (2022). Swarming genetic algorithm: A nested fully coupled hybrid of genetic algorithm and particle swarm optimization. PLoS ONE.

[24] Wei L., Xv S., Li B. (2022). Short-term wind power prediction using an improved grey wolf optimization algorithm with back-propagation neural network. Clean Energy.

[25] Zhang R., Mak S., Dunson D. (2022). Gaussian process subspace prediction for model reduction. SIAM J. Sci. Comput.

[26] Vouros G.A. (2022). Explainable deep reinforcement learning: State of the art and challenges. ACM Comput. Surv.

[27] Newaz N.T., Hanada E. (2023). The Methods of Fall Detection: A Literature Review. Sensors.

[28] Chen M., Wang H., Yu L., Yeung E.H.K., Luo J., Tsui K.-L., Zhao Y. (2022). A Systematic Review of Wearable Sensor-Based Technologies for Fall Risk Assessment in Older Adults. Sensors.

[29] Bezold J., Krell-Roesch J., Eckert T., Jekauc D., Woll A. (2021). Sensor-based fall risk assessment in older adults with or without cognitive impairment: A systematic review. Eur. Rev. Aging Phys. Act..

[30] Alabdulkreem E., Alduhayyem M., Al-Hagery M.A., Motwakel A., Hamza M.A., Marzouk R. (2024). Artificial Rabbit Optimizer with deep learning for fall detection of disabled people in the IoT Environment. AIMS Math..

[31] Krishnamurthi R., Kumar A., Gopinathan D., Nayyar A., Qureshi B. (2020). An Overview of IoT Sensor Data Processing, Fusion, and Analysis Techniques. Sensors.

[32] Howcroft J., Kofman J., Lemaire E.D. (2013). Review of fall risk assessment in geriatric populations using inertial sensors. J. NeuroEng. Rehabil..

[33] Larik R.M., Mustafa M.W., Aman M.N., Jumani T.A., Sajid S., Panjwani M.K. (2018). An Improved Algorithm for Optimal Load Shedding in Power Systems. Energies.

[34] Yang D., Yu Z., Yuan H., Cui Y. (2022). An improved genetic algorithm and its application in neural network adversarial attack. PLoS ONE.

[35] Sánchez-Ibáñez J.R., Pérez-del-Pulgar C.J., García-Cerezo A. (2021). Path Planning for Autonomous Mobile Robots: A Review. Sensors.

[36] Liu H., Lang B. (2019). Machine Learning and Deep Learning Methods for Intrusion Detection Systems: A Survey. Appl. Sci..

[37] Jalal A., Kamal S., Kim D. (2014). A Depth Video Sensor-Based Life-Logging Human Activity Recognition System for Elderly Care in Smart Indoor Environments. Sensors.

[38] Bajaj A., Sangwan O.P. (2019). A systematic literature review of test case prioritization using genetic algorithms. IEEE Access.

[39] Li Y., Lin X., Liu J. (2021). An improved gray wolf optimization algorithm to solve engineering problems. Sustainability.

[40] Lim S.M., Sultan A.B.M., Sulaiman M.N., Mustapha A., Leong K.Y. (2017). Crossover and mutation operators of genetic algorithms. Int. J. Mach. Learn. Comput..

[41] Ozsoydan F.B. (2019). Effects of dominant wolves in grey wolf optimization algorithm. Appl. Soft. Comput..

[42] Teng Z., Lv J., Guo L. (2019). An improved hybrid grey wolf optimization algorithm. Soft Comput..

[43] Akram A., Zafar K., Mian A.N., Baig A.R., Almakki R., AlSuwaidan L., Khan S. (2023). On Layout Optimization of Wireless Sensor Network Using Meta-Heuristic Approach. Comput. Syst. Sci. Eng..

[44] Zhao Z., Chen K., Liu Y., Bao H. (2023). A Large-Scale Sensor Layout Optimization Algorithm for Improving the Accuracy of Inverse Finite Element Method. Sensors.

